# Elevated CO_2_ as a Biostimulatory Approach to Enhance the Nutraceutical Potential of Ginseng

**DOI:** 10.3390/cimb48070676

**Published:** 2026-06-30

**Authors:** Hamad Hussain, Nooral Amin, Imran Ali, Abdul Wakeel Umar, Naveed Ahmad

**Affiliations:** 1 Institute for Safflower Industry Research of Shihezi University/Pharmacy College of Shihezi University/Key Laboratory of Xinjiang Phytomedicine Resource and Utilization, Ministry of Education, Shihezi 832003, China; hammadagarian629@gmail.com (H.H.); aminagric965@gmail.com (N.A.); 2 Department of Agriculture, Faculty of Chemical and Life Sciences, Abdul Wali Khan University Mardan, Mardan 23200, Pakistan; 3 Department of Botany, Kohat University of Science and Technology, Kohat 26000, Pakistan; drimranali@kust.edu.pk; 4 Guangdong-Hong Kong Joint Laboratory for Carbon Neutrality, Jiangmen Laboratory of Carbon Science and Technology, Jiangmen 529199, China

**Keywords:** elevated CO_2_, root biomass, secondary metabolites, molecular mechanisms, carbon–nitrogen balance, *Panax ginseng*

## Abstract

The continued rise in atmospheric carbon dioxide (CO_2_) concentrations presents a strategic opportunity to harness climate change variables within the framework of precision agriculture. Despite the well-established role of elevated CO_2_ (eCO_2_) in enhancing biomass accumulation, its largely underexplored potential to drive the biosynthesis of secondary metabolites represents a more significant and promising avenue of investigation. This review appraises the physiological and molecular mechanisms through which eCO_2_ enrichment redirects metabolic flux toward secondary metabolite biosynthesis, with far-reaching implications for plant productivity and resilience. Special emphasis is placed on critically evaluating the scientific literature to explore how CO_2_-mediated modulation of the carbon–nutrient balance (CNB) can be strategically leveraged to enhance secondary metabolite yields. Moving from observation to application, integrated strategies are proposed to exploit CO_2_ enrichment in advanced bioreactor systems and controlled-environment greenhouses as a means of maximizing bioactive compound production in ginseng. Pinpointing the regulatory sweet spots at which carbon saturation elicits maximum ginsenoside expression opens a promising avenue for engineering ginseng cultivation systems with sustainable potency and superior bioactivity. Though the full molecular architecture of these pathways in Panax awaits elucidation, converging evidence from related plant systems furnishes a credible mechanistic scaffold for future research.

## 1. Introduction

**Figure 1:**
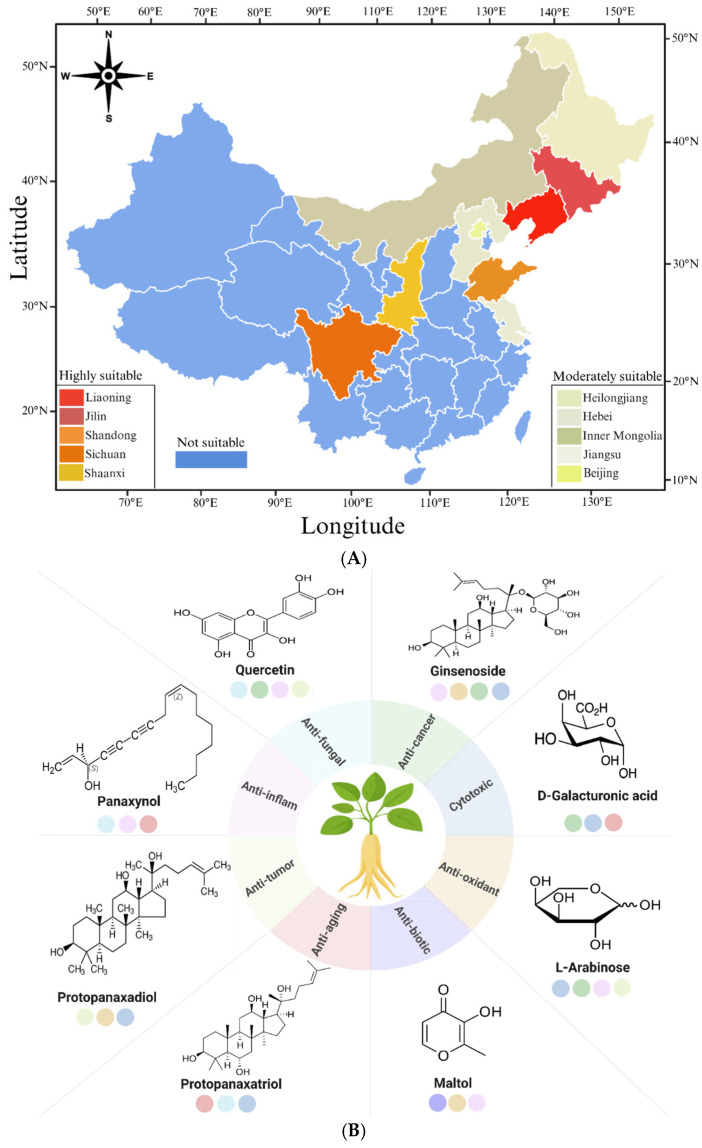
Mapping of regional cultivation suitability for ginseng across China and the associated secondary metabolites responsible for its health properties. (**A**) The maps indicate key areas suitable for ginseng cultivation in China. The regional suitability for ginseng is classified as highly suitable, moderately suitable, and unsuitable for areas all over China. (**B**) The repertoire of secondary metabolites and their associated health properties found in ginseng.

The growing demand for natural therapeutics and functional foods has intensified global interest in medicinal plants as valuable sources of bioactive compounds with diverse pharmacological activities [1,2]. Among these, ginseng (*Panax* spp.) is one of the most economically important and scientifically investigated medicinal plants owing to its long history of use in traditional medicine and its broad spectrum of biological activities [3,4,5]. The pharmacological efficacy of ginseng is primarily attributed to its rich repertoire of specialized metabolites, particularly ginsenosides [6,7,8], a diverse group of glycosylated triterpenoids saponins [6,9,10] (Figure 1). Ginseng-derived products, including extracts, processed formulations, dietary supplements, and functional beverages, are increasingly consumed worldwide because of their demonstrated pharmacological properties [7,8]. Consequently, improving the quality and production efficiency of ginseng has become an important objective for both medicinal plant research and commercial cultivation. Apart from genetic and agronomic approaches, environmental factors are increasingly recognized as important regulators of secondary metabolite biosynthesis in medicinal plants. Among these, atmospheric carbon dioxide (CO_2_) has emerged as a key driver of plant metabolic plasticity. Elevated CO_2_ (eCO_2_) not only enhances photosynthetic carbon assimilation but can also alter carbon allocation patterns, nutrient balance, and biosynthetic pathways associated with specialized metabolite production. Understanding how these physiological and metabolic adjustments influence ginseng phytochemistry may provide new opportunities to improve both crop productivity and medicinal quality under future climatic conditions.

Atmospheric carbon dioxide (CO_2_) has emerged as a key regulator of plant growth and metabolism. Since the industrial revolution, atmospheric CO_2_ concentrations have increased substantially due to anthropogenic activities and now exceed 420 ppm globally [11]. While elevated CO_2_ (eCO_2_) is widely recognized for enhancing photosynthetic carbon assimilation and biomass accumulation through the so-called “CO_2_ fertilization effect” [12,13,14,15,16], accumulating evidence suggests that its influence extends far beyond growth promotion. Elevated CO_2_ can alter the carbon–nitrogen balance, resource allocation patterns, cellular redox status, and metabolic fluxes, thereby influencing the biosynthesis and accumulation of specialized metabolites [17,18]. For example, eCO_2_ has been reported to enhance phenolic and tannin accumulation in cotton [19], increase gallic acid production in *Labisia pumila* [20], modify flavonoid profiles in *Ginkgo biloba* [21], and stimulate antioxidant-related metabolites in strawberry fruits [22]. These findings suggest that eCO_2_ may represent not only a driver of biomass production but also a potential biostimulatory tool capable of enhancing the phytochemical quality and therapeutic value of medicinal plants.

Although considerable research has focused on the effects of eCO_2_ and climate-related factors in major crop species, comparatively limited attention has been given to medicinal plants, particularly ginseng. To date, the available evidence in Panax species remains relatively scarce and is largely derived from cell suspension cultures, adventitious roots, and controlled-environment studies rather than field-grown plants. For example, previous investigations in Panax ginseng suspension cultures demonstrated that eCO_2_ concentrations significantly increased total phenolic, flavonoid, and antioxidant levels by approximately 60%, 30%, and 20%, respectively [23]. Similarly, studies in *Pfaffia glomerata* (Brazilian ginseng) reported enhanced photosynthetic efficiency and substantial modifications in cell wall composition, including increased accumulation of pectin polysaccharides and hemicellulose [24]. While these findings collectively suggest that eCO_2_ has the potential to influence biomass production and secondary metabolism in ginseng and related medicinal species, direct experimental evidence regarding its effects on ginsenoside biosynthesis, metabolic regulation, and molecular signaling pathways in Panax remains limited. Consequently, several mechanistic interpretations concerning carbon allocation, hormonal crosstalk, transcriptional regulation, and metabolic flux reprogramming under eCO_2_ conditions are currently inferred from studies conducted in other medicinal and aromatic plants and should therefore be considered as putative rather than experimentally validated mechanisms in ginseng. These knowledge gaps highlight the need for systematic investigations integrating physiological, molecular, transcriptomic, and metabolomic approaches under realistic cultivation conditions. This review critically evaluates the available evidence on eCO_2_-mediated responses in ginseng, distinguishing direct empirical observations from mechanistic inferences derived from related plant systems. Furthermore, it examines how eCO_2_-driven metabolic reprogramming may enhance secondary metabolite biosynthesis and the therapeutic value of Panax species. Future research directions and cultivation strategies warranting experimental validation for enhancing both the yield and nutraceutical quality of ginseng under changing climatic conditions are also proposed.

### Literature Search Strategy

The literature used in this review was collected through systematic searches of Web of Science, Scopus, PubMed, and Google Scholar databases. Searches were conducted between January and March 2026 and included studies published from 2000 to March 2026. Keywords used individually or in combination included: “elevated CO_2_”, “CO_2_ enrichment”, “*Panax ginseng*”, “*Panax quinquefolius*”, “ginseng”, “ginsenosides”, “secondary metabolism”, “phenolic compounds”, “flavonoids”, “carbon nutrient balance”, “photosynthesis”, “climate change”, and “medicinal plants”. Priority was given to peer-reviewed original research articles and review papers published in English. Studies directly investigating elevated CO_2_ responses in Panax species were preferentially considered. Where direct evidence in Panax was unavailable, relevant findings from other medicinal plant species were included to provide mechanistic context, and such extrapolations are explicitly identified throughout the manuscript.

## 2. Inside out: Elevated CO_2_ as a Biochemical Modulator in Ginseng

Ginseng evolved within temperate forest ecosystems under atmospheric CO_2_ concentrations markedly lower than those of the present day, representing a fundamentally altered growing environment relative to its evolutionary origins [25]. Elevated atmospheric CO_2_ transcends its conventional role in photosynthesis and carbon fixation, triggering broad metabolic reprogramming that reshapes carbon flux, precursor pool availability, and transcriptional regulation of biosynthetic pathways [26,27]. The complex metabolic response elicited by eCO_2_ in ginseng creates new opportunities for exploring its therapeutic potential. The molecular architecture of these pathways remains to be fully resolved in Panax; nevertheless, mechanistic insights derived from related plant systems [28,29] provide a credible framework for future experimental investigation. This section delineates the mechanistic basis by which eCO_2_ functions as a biochemical modulator, redirecting secondary metabolic fluxes in ginseng through discrete molecular regulatory networks.

The increasing level of atmospheric CO_2_ presents both challenges and significant opportunities for the cultivation of medicinal plants, particularly *Panax ginseng*. As a fundamental substrate for photosynthetic reactions, eCO_2_ is well established to enhance primary carbon assimilation by stimulating photosynthesis in ginseng [30]. However, excess CO_2_ may additionally function as a biochemical effector, reprogramming secondary metabolic pathways to enhance the biosynthesis of therapeutically valuable metabolites [23,31]. Corroborating evidence demonstrates that eCO_2_ exposure promotes the selective accumulation of phenolics, flavonoids, and specific ginsenoside saponins in ginseng. For instance, under CO_2_ enrichment ranging from 1% to 5%, *Panax ginseng* cultures showed a significant increase in the amounts of phenolics and antioxidant activity, accompanied by enhanced activities of enzymes like phenylalanine ammonia-lyase (PAL), glucose-6-phosphate dehydrogenase (G6PDH), and polyphenol oxidase (PPO), which regulate the phenylpropanoid pathway and pentose phosphate pathway [23,32]. These findings imply that CO_2_ not only serves as a substrate for photosynthesis but also functions as a key regulator of plant metabolism, influencing carbon allocation and the synthesis of specialized metabolites.

**Figure 2:**
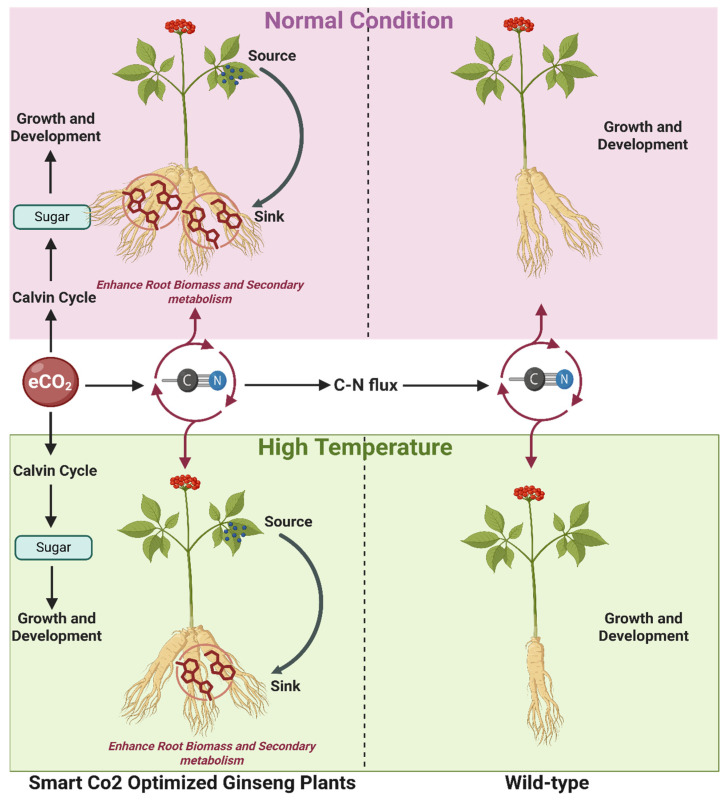
Conceptual model demonstrating the potential physiological and metabolic responses of Panax ginseng to elevated CO_2_ under normal and stressful thermal conditions. The model is based on currently available evidence from suspension culture of Panax species and evolutionary conserved plants and is yet to be fully elucidated in whole-plant ginseng systems. Black arrows in the schematic indicate pathways supported by direct evidence in Panax cell/tissue culture systems; red arrows indicate relationships that are hypothetical or extrapolated from evolutionarily related and model plant species and have not yet been demonstrated in whole-plant Panax. Based on the proposed hypothetical framework, “intelligent CO_2_-fertilized ginseng plants” could be produced via an innovative approach incorporating CO_2_ delivery based on sensing, precise environmental control, and/or CO_2_ responsive genetics. Such plants grow at increased CO_2_ levels (eCO_2_) through an intelligent system that optimizes the carbon uptake of plants according to their needs. This induces activation of the Calvin cycle, resulting in increased sugar content and better source–sink relationships toward roots. Thus, there is an increased production of root biomass and secondary metabolites, even during heat stress. Efficient C-N flow improves plant health and metabolism. By comparison, ginseng grown without such sensing-based CO_2_ management is hypothesized to show a more limited capacity to convert eCO_2_ into root biomass and stress tolerance, although this comparative outcome has not yet been directly tested and is presented here as a conceptual contrast rather than an established finding.

Nevertheless, the CO_2_ concentrations employed in cell and tissue culture systems are often substantially higher than those used in greenhouse or controlled-environment cultivation. Therefore, findings derived from in vitro studies using 1–5% CO_2_ [23] should be interpreted cautiously and should not be directly extrapolated to whole-plant ginseng production under physiologically relevant eCO_2_ conditions (600–1200 ppm). Furthermore, the metabolic changes induced by eCO_2_ are consistent with the carbon–nutrient balance hypothesis, whereby excess carbon generated through enhanced photosynthesis is allocated to the synthesis of secondary metabolites [33,34]. This metabolic reallocation represents a key adaptive mechanism that enhances plant chemical defense. Several studies have reported approximately 50% increases in root phenolic concentrations under supra-optimal CO_2_ enrichment (10,000–25,000 ppm) relative to ambient atmospheric conditions (~400 ppm) [23]. Similar responses have also been documented in other medicinal and aromatic plants, including *Ocimum basilicum* and *Mentha piperita* [35,36], where elevated CO_2_ stimulated the accumulation of bioactive secondary metabolites. Although direct evidence remains limited for the ginseng whole-plant system, these pronounced biochemical alterations observed in ginseng cells and related medicinal species under eCO_2_ suggest considerable potential for enhancing the phytochemical quality and therapeutic value of ginseng (Figure 2). In parallel, CO_2_-assisted supercritical fluid extraction has emerged as an effective technology for recovering high-value phytochemicals from ginseng, yielding extracts with enhanced antioxidant and anti-inflammatory activities [37,38]. Nevertheless, plant responses to elevated CO_2_ are highly context-dependent. Excessively high CO_2_ concentrations, particularly when combined with elevated temperatures, have been reported to suppress biomass accumulation and reduce saponin production in cultured ginseng cells [39]. Consequently, identifying an optimal CO_2_ concentration range is essential for maximizing both growth performance and phytochemical productivity [31]. Further, researchers may also leverage eCO_2_-induced growth promotion in ginseng to increase both biomass accumulation and bioactive metabolite concentration per unit weight. Preliminary studies demonstrated that eCO_2_ levels promote plant growth by stimulating photosynthesis in a number of plant species, including *Panax ginseng*, resulting in increased biomass [40,41,42,43,44]. Yet the downside of using eCO_2_ is the phenomenon of the “dilution effect”, where rapid growth induced by eCO_2_ leads to relatively low production of secondary metabolites [45,46].

To overcome the trade-off between biomass production and phytochemical accumulation, we propose a two-phase CO_2_ management strategy for ginseng cultivation. It is important to note that the CO_2_, nitrogen, and elicitor levels proposed below represent initial experimental parameters intended as a starting point for hypothesis testing, rather than optimized cultivation conditions; their suitability has not yet been empirically determined for Panax. In the first phase (biomass accumulation phase), plants are grown under elevated CO_2_ concentrations (1000–1200 ppm) to maximize photosynthesis and promote vegetative growth and root biomass development. In the second phase (phytochemical induction phase), CO_2_ levels are reduced to 600–800 ppm and combined with elicitors such as methyl jasmonate (MeJA), salicylic acid (SA), and controlled nitrogen limitation to stimulate the biosynthesis of ginsenosides and other bioactive metabolites (Figure 2). This strategy exploits the dual role of CO_2_ in regulating both plant growth and secondary metabolism, providing a non-transgenic approach to improve ginseng productivity and phytochemical quality [37,38]. The resulting increase in bioactive compounds could be further complemented by supercritical CO_2_ extraction technologies to maximize phytochemical recovery. However, this framework is largely extrapolated from evidence in ginseng cell cultures and related plant species. Future studies should determine the optimal CO_2_ transition points, exposure durations, and developmental stages required to balance biomass accumulation with phytochemical production under commercial cultivation conditions.

For future experimental validation, we propose the following experimental parameters, offered as a starting point rather than optimized conditions: (i) Phase 1 (biomass building): whole-plant Panax ginseng grown under 1000–1200 ppm CO_2_ in controlled-environment chambers with optimal nitrogen (5 mM NO_3_^−^), 25/18 °C day/night temperatures, and 12 h photoperiod, with biomass (fresh and dry root weight), chlorophyll content, and leaf gas exchange measured at 4-week intervals; (ii) Phase 2 (phytochemical induction): CO_2_ reduced to 600–800 ppm and combined with MeJA (100 μM foliar spray) and low nitrogen (0.5 mM NO_3_^−^), with ginsenoside profiles (individual Rb1, Rb2, Rc, Rd, Re, Rg1 by HPLC-MS), total phenolics, and key biosynthetic gene expression (HMGCR, FPS, SS, CYP716A47) quantified at harvest. Such a systematic approach will allow direct assessment of whether the dilution effect can be reversed by stage-specific CO_2_ transitions and will provide the first empirical evidence base for optimizing eCO_2_-assisted ginseng cultivation.

## 3. Metabolic Flux Control and Enzymatic Gateways Under CO_2_ Enrichment

Elevated atmospheric CO_2_ is increasingly recognized not merely as a photosynthetic substrate but as a potent biochemical signal capable of orchestrating far-reaching and sophisticated metabolic flux redirection with profound implications for secondary metabolite biosynthesis. Evidence indicates that the biochemical architecture governing secondary metabolism in Panax species is inherently modular, with metabolic flux coordinately regulated by critical gateway enzymes, including phenylalanine ammonia-lyase (PAL), 3-hydroxy-3-methylglutaryl-CoA reductase (HMGR), and farnesyl diphosphate synthase (FPS) [3]. These enzymes control the metabolic entry of carbon flux into phenolics and ginsenoside branches. Studies on closely related plants, e.g., *P. glomerata* and *P. ginseng*, indicate increased activity of PAL and antioxidant enzymes due to eCO_2_, contributing to increased amounts of phenolics while maintaining the cellular oxidation–reduction balance [23,24]. One compelling mechanistic basis for the eCO_2_-driven enhancement of secondary metabolism is the alleviation of rate-limiting metabolic bottlenecks through upregulation of carbon fixation enzyme activities. Although the complete regulatory architecture of these pathways has yet to be fully delineated within the genus Panax, evolutionarily conserved and ectopically expressed mechanisms characterized in model plant systems represent a robust mechanistic scaffold that can be strategically applied to accelerate the elucidation of secondary metabolic regulatory networks in ginseng.

**Figure 3:**
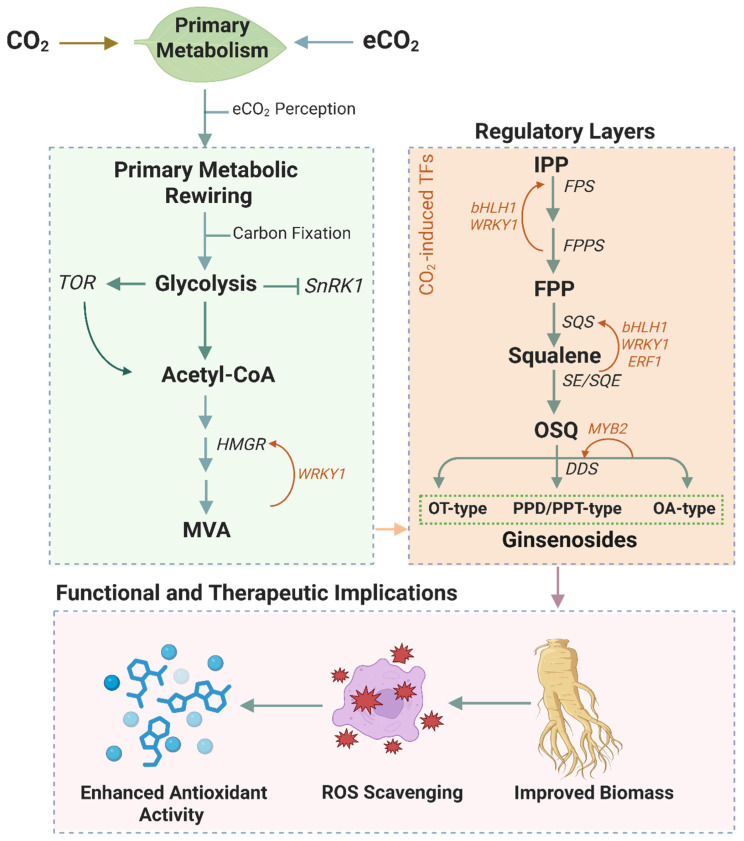
Proposed theoretical model illustrating putative regulatory checkpoints that may contribute to eCO_2_-mediated ginsenoside biosynthesis in Panax species. The model is adopted from evolutionarily conserved and/or model plant systems suggesting that similar mechanisms may contribute to metabolic regulation in ginseng under elevated CO_2_ conditions. Solid green arrows denote enzymatic and biosynthetic steps with direct experimental support in Panax or closely related triterpenoid-producing species; red arrows denote transcription factor–enzyme links and signaling steps that are hypothetical, inferred from model plant systems, and not yet validated in Panax. This schematic framework illustrates how eCO_2_ stimulates primary metabolism, leading to metabolic rewiring and enhanced biosynthesis of ginsenosides in ginseng, alongside functional and therapeutic benefits. Box 1: Primary metabolic rewiring under eCO_2_. At first, the eCO_2_ levels are perceived by the plant, triggering enhanced primary metabolism, including carbon fixation and glycolysis, resulting in increased production of acetyl-CoA. This acetyl-CoA is then channeled through the mevalonate (MVA) pathway, with HMGR (3-hydroxy-3-methylglutaryl-CoA reductase) acting as a key rate-limiting enzyme. Transcription factor WRKY1 is shown to upregulate HMGR expression, promoting flux through the MVA pathway. Box 2: Regulatory layers governing eCO_2_-induced ginsenoside biosynthesis. Downstream of MVA, isopentenyl pyrophosphate (IPP) is synthesized, which is subsequently converted to farnesyl pyrophosphate (FPP) by FPS (farnesyl diphosphate synthase) and FPPS. FPP is then converted to squalene by squalene synthase (SQS), a key branching point in triterpenoid biosynthesis. Further oxidation by SE/SQE (squalene epoxidase) produces 2,3-oxidosqualene (OSQ), the precursor to ginsenosides. eCO_2_-responsive transcription factors, including bHLH1, WRKY1, ERF1, and MYB2, regulate key enzymatic steps (e.g., FPS, SQS, DDS) in this pathway. OSQ is cyclized by either dammarenediol synthase (DDS) or β-amyrin synthase and further modified into different classes of ginsenosides: OT-type (oleanane-type), PPD/PPT-type (protopanaxadiol and protopanaxatriol types), and OA-type (ocotillol-type). Box 3: Functional and therapeutic implications. The enhanced biosynthesis of ginsenosides under elevated CO_2_ conditions leads to beneficial physiological and medicinal outcomes, including improved biomass production of ginseng roots, ROS (reactive oxygen species) scavenging properties, and enhanced antioxidant activity, contributing to potential therapeutic applications.

In model plants, it was observed that elevated concentrations of photoassimilates, particularly sucrose and glucose, under CO_2_ enrichment may act as signaling molecules associated with regulatory pathways such as SnRK1 (SNF1-related kinase 1) and TOR (target of rapamycin) in non-Panax systems (Figure 3). By contrast, direct data on sugar signaling mechanisms and their interaction with CO_2_ in ginseng is limited. However, similar regulatory logic has been documented in the metabolic networks of other plant organisms, which suggests−as a hypothesis still to be tested in Panax−a possible mechanism by which eCO_2_ might enhance secondary metabolism via energy-sensing kinases [47,48]. In addition to increased pools of sugars, the activity of another energy pool, reducing equivalents (NADPH), would be crucial as well. Increased G6PDH activity, the first enzyme entering the oxidative pentose phosphate pathway, has been associated with elevated NADPH pools that are thought to be required for cytochrome P450-mediated hydroxylation reactions in ginsenoside biosynthesis, although this link has not yet been directly demonstrated under eCO_2_ in Panax [4,38]. Not only may an increase in NADPH availability due to eCO_2_ increase fluxes in ginsenoside biosynthesis, but higher carbohydrate synthesis could also provide precursors to form glycosylation substrates, UDP-sugars, necessary for UGTs [49]. Such a biochemical background would facilitate the biosynthetic machinery of enzymes forming triterpenoid saponins with HMGR, DXS (1-deoxy-D-xylulose 5-phosphate synthase), CYP716A47 (P450s), and UGT74AE2, being key biosynthetic nodes potentially targetable via CO_2_ enrichment for transcriptional upregulation [50].

Considering metabolism from the systemic perspective, eCO_2_ may lead to metabolic flux reprogramming not only due to substrate supply changes but also due to reduced feedback inhibition and metabolic homeostatic constraints. Elevated pools of sugar and NADPH are capable of decreasing feedback constraints on PAL and HMGR activities, resulting in increased fluxes in phenylpropanoid and isoprenoid metabolism [51,52]. Consistently, there are similar mechanisms reported in specialized metabolite biosynthesis from different sources. For instance, the increased activity of PAL and geraniol 10-hydroxylase (G10H) promotes vindoline and catharanthine accumulation in *Catharanthus roseus*, illustrating evolutionary conservation in metabolic plasticity favorable for increasing flux through the pathway with increased carbon supply [53]. The use of these findings and implementation of innovations in cultivation practice and pharmaceutical production require the application of novel biotechnology strategies. Current genome editing techniques, such as CRISPR/Cas-based systems, may provide future opportunities for targeted promoter modulation or gene expression enhancement of rate-limiting enzymes involved in ginsenoside biosynthesis, including cytochrome P450s and UGTs. However, these applications are yet to be fully mapped within the genus Panax [54]. Additionally, the design of CO_2_-responsive promoters will allow building future dynamic control systems based on metabolic and cellular signals (i.e., redox or sugar signaling).

A complementary strategy employs the synergistic application of exogenous elicitors, including jasmonic acid or chitosan, in conjunction with CO_2_ enrichment to upregulate transcriptional activity and promote the accumulation of targeted metabolic pools. Elicitors have already proven their capability to stimulate transcriptional cascades and further metabolite biosynthesis [55]. Therefore, the combination of both elicitors and CO_2_ enrichment can result in enhanced gene expression and metabolite content [56]. However, aside from genetic modifications, the elevated level of carbon (sucrose/glucose) and NADPH induced by eCO_2_ can create a metabolic background suitable for the use of engineered scaffolds of metabolic enzymes. According to the review by Bhatnagar-Mathur et al. [57], simultaneous localization of metabolic enzymes involved in biosynthetic pathways such as HMGR, cytochrome P450s, and UGTs by means of protein scaffolding (domain interaction) produces artificial metabolons that can increase substrate channeling and catalytic efficiency of reactions resulting in enhanced product yield by preventing competitive reactions and diffusion loss. Combining such enzymatic constructs with increased levels of precursors and cofactors in the reaction mixture due to eCO_2_ could provide even further enhancement of biosynthesis flux.

To provide a clearer evidence hierarchy, Table 1 presents a comparative summary of key studies examining the effects of elevated CO_2_ on secondary metabolites across Panax species, related medicinal plants, crop species, and model plants, distinguishing direct findings in whole-plant systems from cell/tissue culture studies and extrapolations from other species.

## 4. A Step Forward Toward CO_2_-Induced Therapeutic Landscapes in Ginseng

**Figure 4:**
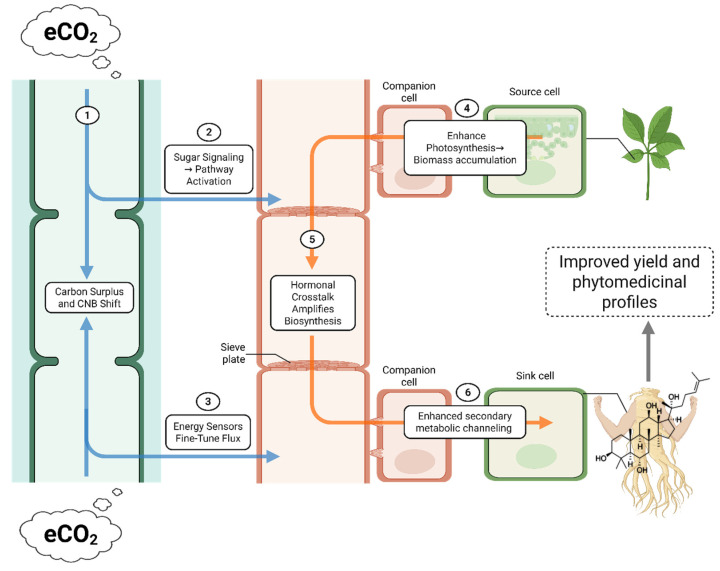
Carbon surplus and CNB shift. By enhancing photosynthesis, eCO_2_ produces a situation where carbon is abundant while nitrogen is reduced, which leads to a greater preference for carbon metabolism products (e.g., ginsenosides, flavonoids). Step 1: Increased levels of soluble sugars serve as signals that upregulate the expression of genes encoding for enzymes involved in secondary metabolism (e.g., HMGCR, FPS, SS; PAL axis). 2: The energy/redox status activates key regulators (e.g., TOR/SnRK1 system logic), coordinating growth–defense relationships such that, when there is excess carbon, resources are channeled toward specialized metabolites. 3: eCO_2_ shifts the balance between JA/SA/ABA, sensitizing the defense machinery, while JA-like signals stimulate the expression of the ginsenoside pathway and its related transport/processing mechanisms. Step 1 is supported by direct evidence of sugar-responsive biosynthetic gene expression in stressed Panax root tissue; Steps 2 and 3 (TOR/SnRK1 signaling logic and JA/SA/ABA crosstalk) are hypothetical extensions inferred from model and related plant species and remain to be tested directly in Panax under eCO_2_.

In *Panax ginseng*, elevated CO_2_ operates as a pleiotropic metabolic driver, simultaneously influencing carbon assimilation efficiency, defense signaling cascades, and secondary metabolite biosynthetic pathways that collectively determine its therapeutic value. Drawing on both classical frameworks and contemporary experimental advances, this section delineates the mechanistic landscape through which eCO_2_ shapes the qualitative phytochemical profile of ginseng (Figure 4).

### 4.1. Carbon–Nutrient Balance Hypothesis (CNB)

The carbon–nutrient balance hypothesis (CNB) proposes that eCO_2_-driven enhancement of photosynthetic carbon fixation generates a carbohydrate surplus [59,60], which is subsequently channeled into carbon-rich secondary metabolites, including phenolics and flavonoids that fulfill key defense and signaling roles [61]. In *P. ginseng*, this implies that eCO_2_ environments may augment the biosynthesis of carbon-intensive triterpenoid saponins, particularly ginsenosides, predominantly in carbohydrate-rich root tissues [32,58]. Meta-analytical evidence across plant species consistently demonstrates eCO_2_-associated increases in total flavonoids and tannins, supporting a broadly conserved pattern of defense metabolite upregulation under elevated carbon conditions [62]. Notwithstanding these carbon-driven gains, the CNB hypothesis explicitly recognizes a stoichiometric nutrient trade-off, wherein elevated carbon availability dilutes tissue nitrogen concentrations, imposing constraints on resource partitioning between primary growth and secondary defense metabolism [63]. Together, these dynamics indicate that the translation of eCO_2_-driven carbon gains into enhanced secondary metabolite accumulation in ginseng is strongly contingent upon soil nutrient availability and tissue-specific allocation.

### 4.2. Photosynthetic Enhancement and Sugar-Mediated Signaling

Photosynthetic enhancement under eCO_2_ conditions represents one of the most immediate and well-documented physiological responses in plants. The resulting increase in photoassimilate production fundamentally alters the cellular carbon economy, with soluble sugars emerging as central mediators linking enhanced carbon fixation to downstream secondary metabolic reprogramming in ginseng. Elevated CO_2_ stimulates net photosynthesis, driving the accumulation of soluble sugars, including glucose and sucrose, which function both as biosynthetic substrates and signaling molecules regulating secondary metabolism [64]. Sugar-mediated regulation of phenylalanine ammonia-lyase (PAL) activity governs phenylpropanoid biosynthesis, representing a critical regulatory node in the production of defensive phenolics and flavonoids [65,66]. In *P. ginseng*, despite limited direct eCO_2_ evidence, soluble sugar accumulation has been positively associated with transcriptional upregulation of the ginsenoside biosynthetic genes encoding HMG-CoA reductase (HMGCR) and squalene synthase (SS), particularly under stress-induced conditions [67,68], implicating sugar signaling as a potential priming mechanism for ginsenoside biosynthesis under eCO_2_. Recent molecular evidence further establishes that root sugar signaling coordinates carbon fixation and secondary biosynthesis through integrated transcriptional and enzymatic regulatory networks [64]. Consequently, eCO_2_-mediated soluble sugar accumulation in ginseng root tissues represents a convergent biosynthetic and signaling nexus for the enhanced production of secondary metabolites.

### 4.3. Hormonal Crosstalk and Defense Signaling

Phytohormonal signaling networks represent a sophisticated regulatory interface through which plants transduce environmental signals into coordinated secondary metabolic responses [69,70]. The potential for eCO_2_ enrichment to interact with jasmonic acid (JA), salicylic acid (SA), and abscisic acid (ABA) signaling cascades in *P. ginseng* constitutes a mechanistically compelling yet largely uncharacterized dimension of CO_2_-mediated secondary metabolic regulation. Notably, exogenous MeJA application in adventitious root cultures of *P. ginseng* elicits a significant 5.5–9.7-fold induction of ginsenoside biosynthesis through the coordinated activation of oxidative and defense-related pathways [71]. This demonstrates the potent regulatory influence of jasmonate signaling on ginsenoside accumulation. Nevertheless, although the full molecular architecture of these regulatory pathways under eCO_2_ conditions remains incompletely understood in Panax, evidence from related plant systems offers a credible mechanistic scaffold for future experimental investigation. The potential modulation of JA, SA, and ABA signaling and their associated crosstalk networks by eCO_2_ in ginseng therefore constitutes a critical and unresolved research priority. Accumulating evidence from other plant species indicates that eCO_2_ conditions can meaningfully reshape hormonal interactions among ABA, SA, and JA, with significant downstream implications for secondary metabolite biosynthesis [72],. However, direct extrapolation of these findings to *P. ginseng* remains scientifically premature without rigorous species-specific experimental validation. To address this critical knowledge gap, future research should prioritize direct quantification of phytohormone levels and comprehensive profiling of hormone-responsive gene expression in *Panax* species under eCO_2_ conditions, ultimately clarifying the role of hormonal crosstalk in mediating eCO_2_-driven secondary metabolic enhancement in ginseng.

### 4.4. Oxidative Signaling and Antioxidant Metabolites

Reactive oxygen species (ROS) signaling represents an evolutionarily conserved regulatory interface through which plants integrate environmental stress perception with secondary metabolic reprogramming. Elucidating the dynamic interplay between eCO_2_-modulated ROS homeostasis and redox-sensitive biosynthetic regulatory networks offers a mechanistically significant yet largely uncharacterized regulatory axis for optimizing ginsenoside accumulation in ginseng. While eCO_2_-mediated photosynthetic stimulation generally attenuates cellular ROS accumulation, environmentally induced variability precipitates localized oxidative bursts that function as priming signals for secondary metabolite biosynthesis and defense activation [27,73,74]. Corroborating this premise, water-stressed *P. ginseng* root tissues demonstrate concurrent increases in H_2_O_2_ levels, NADPH oxidase activity, and the activities of key antioxidant enzymes, including SOD, CAT, POD, APX, and GR, alongside coordinate upregulation of ginsenoside biosynthetic enzyme activities encompassing HMGCR, FPS, and SS [68]. These mechanistic observations, obtained under water-deficit rather than eCO_2_ conditions, suggest oxidative signaling as a plausible regulatory node linking environmental stress perception to ginsenoside biosynthetic activation, although whether the same oxidative mechanisms are engaged specifically by eCO_2_ remains to be tested directly in Panax. Critically, under eCO_2_ conditions, mild localized oxidative signals are hypothesized to engage redox-sensitive transcription factors and downstream biosynthetic enzymes, which would represent a possible mechanistic conduit for eCO_2_-driven enhancement of ginsenoside and antioxidant metabolite production in ginseng. If confirmed experimentally, redox-mediated regulatory control could represent one of several determinants of the medicinal phytochemical profile of ginseng under changing atmospheric CO_2_ conditions; at present, this remains a testable hypothesis rather than an established mechanism.

### 4.5. Species and Tissue-Specific Detail in Ginseng

The metabolic consequences of eCO_2_ enrichment in ginseng are fundamentally governed by the interactive effects of CO_2_ concentration, tissue identity, and developmental stage, collectively precluding simplistic generalizations regarding eCO_2_-mediated secondary metabolic responses. Delineating these species- and tissue-specific regulatory parameters is therefore a prerequisite for establishing a mechanistically coherent and experimentally tractable framework for eCO_2_-mediated ginsenoside enhancement. Studies have shown that eCO_2_-mediated responses in ginseng are strongly modulated by CO_2_ concentration, tissue type, and developmental stage. For example, ginseng cell suspension cultures demonstrate enhanced biomass production at moderate CO_2_ concentrations (~1%). However, saponin biosynthesis is sharply suppressed at elevated concentrations (2.5–5%), with saponin content reductions of up to 50% documented [31,32], collectively delineating a critical optimal CO_2_ window with important implications for cultivation management. Complementing this, fibrous roots demonstrate markedly higher ginsenoside biosynthetic enzyme activity relative to main roots under stress conditions [68], firmly establishing tissue specificity as a critical variable in evaluating ginseng responses to eCO_2_ enrichment. Within the optimal CO_2_ range, moderate eCO_2_ elevation (600–800 ppm) is projected to enhance ginsenoside accumulation in fibrous roots through the coordinated synergism of sugar signaling, oxidative priming, and hormonal crosstalk converging on biosynthetic gene activation. Broader meta-analytical evidence across plant species also corroborates that eCO_2_ reliably stimulates flavonoid and tannin biosynthesis, while lignin and total phenolic responses exhibit considerable species- and tissue-dependent variability [75]. Contemporary literature further underscores that eCO_2_, acting synergistically with global warming and associated climatic variables, orchestrates comprehensive metabolic reprogramming with the potential to enhance secondary metabolite production and augment the medicinal value of ginseng [76]. Although ginsenosides represent triterpenoid saponins rather than phenolic compounds, the broadly conserved eCO_2_-driven enhancement of defense-related secondary metabolite accumulation is mechanistically consistent with the predicted reallocation of carbon resources and metabolic regulatory reprogramming.

## 5. Concentration-Dependent and Ecological Context of Elevated CO_2_ Responses

The physiological and metabolic consequences of eCO_2_ enrichment in plants are neither uniform nor linearly proportional to CO_2_ concentration but are instead governed by a complex interplay of concentration thresholds, exposure duration, and ecological context. For long-lived medicinal species such as Panax, accurately characterizing these concentration-dependent and ecologically contextualized dimensions is not merely scientifically important but practically indispensable for designing effective and sustainable eCO_2_-based cultivation strategies. eCO_2_-mediated physiological and metabolic responses in plants, including medicinal species such as Panax, are broadly recognized as concentration-dependent and inherently non-linear [12]. At moderate CO_2_ enrichment levels (approximately 600–1200 ppm), numerous studies documented enhanced photosynthetic efficiency, elevated carbon assimilation, and improved biomass accumulation, attributable to Rubisco carboxylation enhancement and suppression of photorespiration [77,78,79,80]. These photosynthetic gains generally expand the carbon skeleton availability for secondary metabolic pathways, including triterpenoid biosynthesis [81]. Conversely, prolonged eCO_2_ exposure frequently induces photosynthetic acclimation, manifested as reductions in Rubisco content, impaired nitrogen assimilation, and transcriptional downregulation of photosynthetic genes [18,80]. This acclimation response, driven by sink limitation and carbohydrate feedback inhibition, progressively restricts carbon fixation capacity [82]. In medicinal plants, such feedback-driven constraints may indirectly limit secondary metabolite accumulation by disrupting the carbon–nitrogen metabolic balance. Carbon–nutrient trade-offs constitute a fundamental metabolic constraint under eCO_2_ conditions. Concurrent with rising carbon assimilation, plant tissue nitrogen concentrations frequently decline as a consequence of dilution effects and diminished nitrate assimilation efficiency [83]. This stoichiometric imbalance compromises enzymatic processes dependent on nitrogen-rich cofactors, thereby constraining key secondary metabolic pathways, including terpenoid biosynthesis [82].

From an ecological standpoint, a fundamental distinction must be maintained between controlled-environment CO_2_ enrichment systems and real-world atmospheric CO_2_ projections. Controlled systems characteristically employ short-term or supra-ambient CO_2_ concentrations designed to elicit maximal physiological responses. In contrast, field conditions entail prolonged eCO_2_ exposure under dynamic temperature, water availability, and nutrient constraints [80]. Free-Air CO_2_ Enrichment (FACE) investigations consistently reveal that plant physiological responses under field conditions are substantially attenuated relative to those in controlled-environment studies, reflecting the constraining influence of environmental co-limitation [77]. Genotype- and species-specific variability further modulates eCO_2_ responsiveness in a pronounced manner. Distinct genotypes demonstrate characteristic differences in carbon allocation strategies, stomatal conductance regulation, and acclimation capacity under eCO_2_ conditions [12]. This genotypic variability is particularly well documented in medicinal and secondary metabolite-producing plants, where biosynthetic output is strongly genotype-dependent [83]. Taken together, these findings establish that eCO_2_ responses in plants are governed by a multifactorial interaction of CO_2_ concentration, exposure duration, nutrient availability, and environmental context. The extrapolation of controlled-environment findings to field-scale medicinal plant production must therefore be approached with considerable circumspection, particularly for long-lived perennial species such as Panax.

## 6. Current Gaps and Opportunities for Ginseng Yield and Quality Improvement Under Elevated CO_2_

Ginseng, particularly *Panax ginseng* and *Panax quinquefolius*, is one of the most valuable medicinal plants worldwide due to its pharmacologically active constituents, mainly ginsenosides, phenolics, and polysaccharides. These secondary metabolites contribute to ginseng’s antioxidant, anti-inflammatory, and adaptogenic properties. However, the biosynthesis of these compounds is sensitive to environmental conditions, including atmospheric CO_2_ levels. Although some initial research has been conducted, the influence of elevated CO_2_ on ginseng metabolism remains insufficiently understood. Previous findings suggest that elevated CO_2_ concentrations may stimulate root development, improve photosynthetic efficiency, and enhance total phenolic and flavonoid production [23]. Despite these initial findings, several important gaps remain:

1. There is limited understanding of how elevated CO_2_ modulates the transcriptional regulation of genes involved in ginsenoside biosynthesis.
2. The dose–response relationship and long-term effects of CO_2_ exposure on ginseng metabolism remain largely unknown.
3. Most studies focus mainly on roots, while leaves and stems may also contribute to or be affected by altered metabolic processes.
4. The combined effects of CO_2_ with light, temperature, and nutrient regimes on ginseng metabolite profiles are poorly studied.
5. Different Panax genotypes may respond differently to CO_2_, but comparative studies are yet to be investigated.
6. The proposed two-phase CO_2_ enrichment model requires empirical validation to determine whether early-stage CO_2_ enrichment can promote biomass accumulation, followed by later-stage regulation.

These gaps also provide several opportunities for improving ginseng yield and quality under controlled cultivation systems:

1. Incorporating CO_2_ enrichment in greenhouse or vertical farming systems could enhance biomass and selectively increase desired metabolites.
2. Understanding CO_2_-responsive genes and pathways may support metabolic engineering or CRISPR-based strategies to improve ginsenoside biosynthesis under elevated CO_2_.
3. Identification of CO_2_-responsive ginseng genotypes could enable breeding of CO_2_-efficient cultivars with improved quality and yield.
4. Monitoring metabolite dynamics under elevated CO_2_ may allow fine-tuning of harvest schedules to achieve maximum bioactive compound accumulation.
5. Future experiments should test stage-specific CO_2_ management strategies, including the two-phase model, to balance biomass production with medicinal quality.

## 7. Conclusions and Future Directions

Elevated atmospheric CO_2_ presents both an opportunity and a challenge for improving ginseng production and quality through the modulation of secondary metabolism. Existing studies suggest that moderate CO_2_ enrichment can stimulate biomass accumulation and may influence phenolic compounds, while its effects on ginsenoside content remain complex and dose-dependent. Therefore, future studies should directly examine *Panax* species under different CO_2_ concentrations, exposure periods, growth stages, and cultivation conditions. Particular attention should be given to individual ginsenosides, photosynthesis, carbon allocation, hormone regulation, and key genes involved in ginsenoside biosynthesis. Practical cultivation studies are also needed to determine suitable CO_2_ levels in combination with light, temperature, water, and nutrient management. Overall, integrating physiology, molecular biology, metabolomics, and agronomic validation will be essential for safely using elevated CO_2_ to improve ginseng yield and medicinal quality.

## Figures and Tables

**Table 1:** Comparative summary of published studies on the effects of elevated CO_2_ (eCO_2_) on secondary metabolite biosynthesis across Panax species, related medicinal plants, crop species, and model plants discussed in this review. Studies are organized by species and include information on CO_2_ concentration applied, experimental system, exposure duration, tissue type examined, metabolite class investigated, and the main findings reported. Exposure durations are reported as exact periods where stated in the original publication; where only a qualitative descriptor was reported (e.g., a single field season), this is noted accordingly. The final column (Evidence Type) classifies each entry according to its relevance to whole-plant Panax systems: "Direct" entries represent studies conducted directly in Panax species and are further distinguished by experimental system, whole-plant Panax studies (the most directly translatable) versus Panax in vitro/cell and tissue culture studies (which often employed supra-ambient CO_2_ concentrations not representative of field or greenhouse cultivation). "Extrapolated" entries are findings from related medicinal plants, crop species, or model organisms not yet confirmed in Panax under any system.

Species	CO_2_ Conc.	Experimental System	Exposure Time	Tissue Type	Metabolite Class	Main Findings	Evidence Type	Reference
*Panax ginseng*	1–5% (10,000–50,000 ppm)	Cell suspension culture	4 weeks	Root-derived callus	Phenolics, flavonoids, antioxidants	+60% phenolics, +30% flavonoids, +20% antioxidants; elevated PAL, G6PDH, PPO activity	Direct (in vitro; supra-ambient CO_2_)	[23]
*Panax ginseng*	0.5–5% CO_2_	Root suspension culture	4 weeks	Root cells	Saponins (ginsenosides), antioxidant enzymes	Saponin biosynthesis declines at >2.5% CO_2_; optimal at ~1%; increased antioxidant enzyme activity	Direct (in vitro; supra-ambient CO_2_)	[32]
*Panax japonicus*	800 ppm	Controlled environment (whole plant)	Single growing season (~5–6 months)	Root	Saponins, C/N metabolites	Altered C/N balance; increased saponin accumulation in roots	Direct (whole plant; near-ambient eCO_2_)	[58]
*Pfaffia glomerata* (Brazilian ginseng)	2× ambient (~800 ppm)	In vitro shoot culture	4 weeks	Shoot/leaf	Cell wall polysaccharides (pectin, hemicellulose)	Enhanced photosynthetic efficiency; altered cell wall composition with increased pectin and hemicellulose	Related species (in vitro)	[24]
*Ocimum basilicum*/*Mentha piperita*	700–800 ppm	Controlled environment (greenhouse)	8 weeks	Whole plant (leaf/shoot)	Phenolics, terpenes, antioxidants	Global metabolic changes; improved antioxidant and antimicrobial activities	Other medicinal herbs	[35]
*Gossypium hirsutum* (cotton)	700 ppm	Greenhouse (transgenic and WT)	6 weeks	Leaf	Phenolics, tannins, gossypol	Increased phenolic and tannin allocation under eCO_2_; interaction with N availability	Crop model	[19]
*Labisia pumila*	800 ppm	Controlled environment (whole plant)	15 weeks	Leaf	Phenolics, flavonoids	Increased total phenolics, flavonoids, and PAL activity under eCO_2_	Medicinal plant	[20]
*Ginkgo biloba*	700 ppm	Open-top chamber (whole plant)	1 growing season (~5 months)	Leaf	Flavonoids (quercetin, kaempferol derivatives)	Modified foliar flavonoid profiles under eCO_2_ and O_3_; interaction with oxidative stress	Medicinal tree	[21]
*Fragaria ananassa* (strawberry)	~550–700 ppm (FACE)	FACE field experiment	1 growing season (FACE, ~4 months)	Fruit	Anthocyanins, ascorbate, antioxidants	Elevated antioxidant compound content in field-grown fruits under FACE conditions	Crop/fruit (FACE field data)	[22]
*Gynostemma pentaphyllum*	800 ppm	Controlled environment + elevated temperature	Weeks–months	Whole plant (leaf)	Gypenosides, phenolics, antioxidants	eCO_2_ partially offset heat stress effects; altered gypenoside and antioxidant profiles	Medicinal plant/adaptogen	[39]
*Arabidopsis thaliana*	700–1000 ppm	Controlled-environment growth chamber	6 weeks (full life cycle)	Whole plant (leaf)	Starch, sugars, structural changes, N compounds	Physiological, biochemical, and structural leaf changes; key model for CO_2_ signaling via SnRK1/TOR	Model plant (mechanistic framework)	[41]
*Brassica oleracea* (cauliflower/cabbage)	800 ppm + drought	Controlled environment (whole plant)	Weeks	Whole plant (leaf/head)	Glucosinolates, phenolics, carotenoids	eCO_2_ modulated secondary metabolites; interaction with drought stress altered glucosinolate profiles	Crop plant	[42]
*Glycine max* (soybean)	550–800 ppm	Controlled environment (whole plant)	Full growing season (~16 weeks)	Leaf	Starch, non-structural carbohydrates, N content	Photosynthetic downregulation; increased non-structural carbohydrates; decreased leaf N content—supports CNB hypothesis	Crop model for CNB	[43]

## Data Availability

No new data were created or analyzed in this study. Data sharing is not applicable to this article.
